# Current and future pharmacological therapies for NAFLD/NASH

**DOI:** 10.1007/s00535-017-1415-1

**Published:** 2017-12-16

**Authors:** Yoshio Sumida, Masashi Yoneda

**Affiliations:** Division of Hepatology and Pancreatology, Department of Internal Medicine, Aichi Medical University, Nagakute, Aichi 480-1195 Japan

**Keywords:** Diabetes, Hepatic fibrosis, NASH, SGLT2 inhibitor, GLP-1 receptor agonist

## Abstract

Nonalcoholic fatty liver disease (NAFLD) is the most prevalent liver disease worldwide, and there is no approved pharmacotherapy. The efficacy of vitamin E and pioglitazone has been established in nonalcoholic steatohepatitis (NASH), a progressive form of NAFLD. GLP-1RA and SGLT2 inhibitors, which are currently approved for use in diabetes, have shown early efficacy in NASH, and also have beneficial cardiovascular or renal effects. Innovative NASH therapies include four main pathways. The first approach is targeting hepatic fat accumulation. Medications in this approach include modulation of peroxisome proliferator-activator receptors (e.g., pemafibrate, elafibranor), medications targeting farnesoid X receptor axis [obeticholic acid; OCA)], inhibitors of de novo lipogenesis (aramchol, ACC inhibitor), and fibroblast growth factor-21 analogues. A second target is oxidative stress, inflammation, and apoptosis. This class of drug includes apoptosis signaling kinase 1 (ASK1) inhibitor and emricasan (an irreversible caspase inhibitor). A third target is intestinal microbiomes and metabolic endotoxemia. Several agents are in ongoing trials, including IMMe124, TLR4 antagonist, and solithromycin (macrolide antibiotics). The final target is hepatic fibrosis, which is strongly associated with all-cause or liver-related mortality in NASH. Antifibrotic agents are a cysteine–cysteine motif chemokine receptor-2/5 antagonist (cenicriviroc; CVC) and galectin 3 antagonist. Among a variety of medications in development, four agents such as OCA, elafibranor, ASK1 inhibitor, and CVC are currently being evaluated in an international phase 3 trial for the treatment of NASH. Within the next few years, the availability of therapeutic options for NASH will hopefully curb the rising trend of NASH-related diseases.

## Introduction

Nonalcoholic fatty liver disease (NAFLD) is the most prevalent chronic liver disease. One-fourth of the adult population is now suffering from NAFLD worldwide [1, 2]. Nonalcoholic steatohepatitis (NASH), the aggressive form of NAFLD, can progress to cirrhosis and hepatocellular cancer (HCC) and is rapidly becoming the leading cause for end-stage liver disease or liver transplantation [3]. In Japan, liver-related diseases, such as cirrhosis and HCC, are now the third leading cause of death in type 2 diabetes mellitus (T2DM) [4], which is closely associated with NAFLD. It is estimated that the prevalence of diagnosed NASH will reach 18 million by 2027 in US, Japan, and EU 5 (England, France, Germany, Italy, and Spain). Lifestyle interventions, such as dietary caloric restriction and exercise, currently the cornerstone of therapy for NASH/NAFLD, can be difficult to achieve and maintain, underscoring the dire need for pharmacotherapy. However, there are no approved pharmacotherapies for NASH/NAFLD. This review presents the agents currently used in managing NASH/NAFLD and their pharmacologic targets. It also provides an overview of NAFLD agents currently under development.

### Currently recommended pharmacotherapies in the practice guidelines from US, Europe, and Japan

Evidence-based practice guidelines for the management of NASH/NAFLD were published by the American Association for the Study of Liver Disease (AASLD) in 2012 [5], the Japan Society of Gastroenterology (JSG)–Japan Society of Hepatology (JSH) in 2014 [6], and the European Association for the Study of the Liver (EASD)–European Association for the Study of Diabetes (EASL)–European Association for the Study of Obesity (EASO) in 2016 [7]. The AASLD recently proposed NAFLD “guidance” to help clinicians understand and implement the most recent evidence [8]. In summary, pioglitazone and vitamin E are now recommended as pharmacotherapies for biopsy-proven NASH patients with and without diabetes, respectively, although long-term efficacy or safety should be established (Table 1) [5–8].

**Table 1:** The summary of recommended pharmacotherapies for NASH/NAFLD in guidelines or guidance

	AGA/AALSLD (2012)	JSG/JSH (2014)	EASL/EASD/EAO (2016)	AASLD guidance (2017)
Vitamin E	First-line therapy for biopsy-proven NASH without diabetes and cirrhosis (800 mg/day)	Recommended	Not firmly recommended, but could be used	May be considered in biopsy-proven NASH without diabetes and cirrhosis (800 mg/day) Discuss benefit and risk with patients
UDCA	Not recommended	Not recommended	Not mentioned in detail	Not recommended
Pioglitazone	Can be used in patients with biopsy-proven NASH	Recommended in NASH with insulin resistance	Not firmly recommended, but could be used	Can be used in patients with biopsy-proven NASH Discuss benefit and risk with patients
Metformin	Not recommended as a specific treatment for NASH	Not recommended as a specific treatment for NASH	Insufficient evidence	Not recommended as a specific treatment for NASH
GLP-1RA	Not mentioned	Not mentioned	Not mentioned	Premature as a specific treatment for NASH
ω3 fatty acid	May be considered in NAFLD with hypertriglyceridemia	Not mentioned	Reduced lipid in plasma and liver, but no evidence related to NASH	Not recommended as a specific treatment for NASH May be considered in NAFLD with hypertriglyceridemia
Statin	Can be used to treat dyslipidemia	Recommended for hypercholesterolemia	Can be used to reduce LDL-C and prevent cardiovascular risk	Can be used to treat dyslipidemia Should be avoided in decompensated cirrhosis
Pentoxifylline	Not mentioned	Recommended, but commercially unavailable in Japan	Not mentioned	Not mentioned
OCA	Not mentioned	Not mentioned	Not mentioned	Off-label use not recommended (approved for PBC in USA)

### Whom to treat

**Fig. 1:**
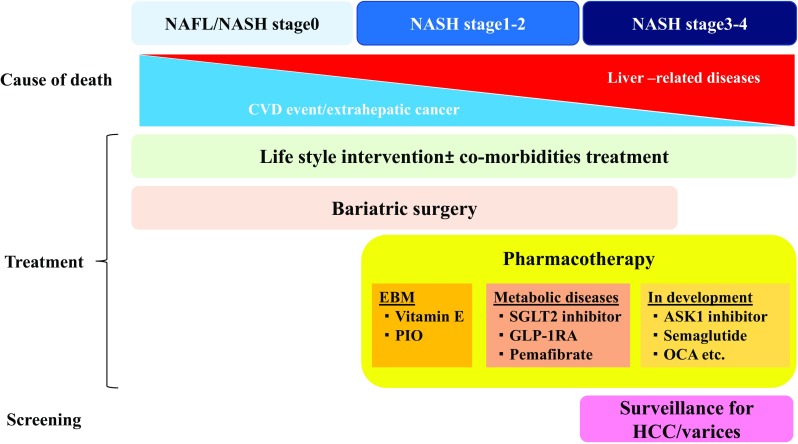
Fibrosis stage-based treatment algorithm for NASH/NAFLD

Dietary changes and lifestyle modifications are now the first-line therapy for patients with NASH. Body weight reduction cannot be achieved in a lot of patients. The most difficult question to answer is what will be the target population of NAFLD/NASH pharmacologic treatment. According to the practice guideline from Europe proposed in 2016, pharmacotherapies should be considered for NASH patients with fibrosis stage 2 or higher and with early stage fibrosis with high risk of fibrosis progression (older age, diabetes, metabolic syndrome, increased ALT, and high necroinflammatory activity) [5]. According to a meta-analysis evaluating five adult NAFLD cohort studies, the presence of advanced fibrosis (stage 2 or more) is the most important predictor of liver-related mortality in NAFLD patients [9]. The AASLD guidance also suggests that pharmacotherapies aimed primarily at improving liver disease should be limited to patients with NASH and fibrosis [8]. Therefore, patients with non-aggressive type of NAFLD (NAFL or NASH stage 0) does not require liver-specific treatments, although the prevention of cardiovascular or renal diseases are strategically essential in them (Fig. 1).

### How to treat (Fig. 2)

#### Antioxidants/hepatoprotective drugs

*Vitamin E* Oxidative stress has been implicated to have an important role in the progression of NASH [10, 11]. Vitamin E is well known as a free radical scavenger, and has been expected for the treatment of NASH. We previously reported that vitamin E treatment for 1 year reduced serum transaminase activities as well as transforming growth factor-beta1 in adult NASH patients who were refractory to dietary intervention [12, 13]. In pioglitazone versus vitamin E versus Placebo for the Treatment of Nondiabetic Patients with Nonalcoholic Steatohepatitis (PIVENS) trial, vitamin E (800 mg/day) is superior to placebo for the improvements of NASH histology in adults NASH without diabetes and cirrhosis [14]. According to a random-effects model analysis of the five studies, vitamin E significantly reduced serum hepatobiliary enzymes, hepatic steatosis, inflammation, and hepatocellular ballooning compared with the control group [15]. In those studies, however, fibrosis improvement was not confirmed. In Japan, long-term vitamin E treatments (300 mg/day) for more than 2 years can ameliorate hepatic fibrosis in NASH patients, especially in those whose serum transaminase activities and insulin resistance can be improved [16]. This result has suggested that metabolic factors should be controlled even when vitamin E is administrated. Although vitamin E is now recommended only for biopsy-proven NASH patients without diabetes on the basis of PIVENS trial, it is associated with histological improvement regardless of diabetic status [17]. However, the primary concern regarding vitamin E for NASH treatment has been the potential for toxicity with long-term or high-dose use. Vitamin E treatment may increase all-cause mortality [18], prostatic cancer (SELECT trial) [19], and hemorrhagic stroke [20], although several conflicting results exist. When vitamin E is administrated for NASH, treatment with lower dose (300–400 mg/day rather than 800 mg) of its agent should be considered [17].

**Fig. 2:**
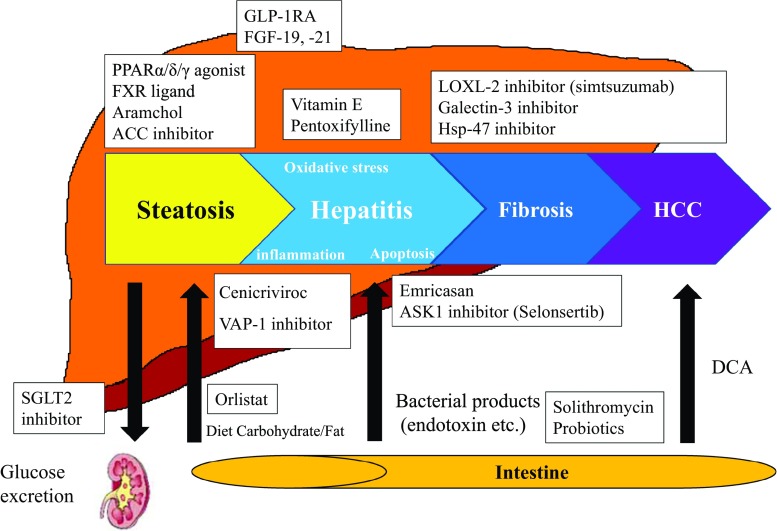
Targets of upcoming therapies for NASH/NAFLD

*Glutathione (GSH)* Glutathione (GSH), l-glutamyl-l-cysteinyl-glycine, is a tripeptide present in every cell of the human body, and also has an anti-oxidative effect. A pilot study found that oral administration of GSH (300 mg/day) for 4 months can decrease ALT levels and hepatic steatosis in Japanese NAFLD patients, in those without severe fibrosis or uncontrolled diabetes. Large-scale clinical trials are needed to verify its efficacy [21].

*UDCA* Ursodeoxycholic acid (UDCA), which is covered by health insurance for chronic liver diseases in Japan, is known to have anti-oxidative efficacy [22]. According to a large, multicenter RCT, standard dose of UDCA has no effects on liver histology in NASH [23], although some studies suggest that high dosage of this agent may show a favorable effect. Currently, UDCA is not recommend for NASH treatment in the guidelines [5–8].

#### Peroxisome proliferator-activated receptor (PPAR) agonists (Fig. 3)

*PPARγ* Two randomized, double-blind, placebo-controlled trials (RDBPCT) have shown that pioglitazone [peroxisome proliferator-activated receptor gamma (PPARγ) agonist] significantly ameliorated steatosis and necroinflammation compared to placebo in diabetic NASH [24, 25]. Recently, a 3-year study in 101 NASH patients with prediabetes/T2DM (an 18-month RCT, followed by an 18-month open-label phase with pioglitazone treatment) confirmed its long-term safety and efficacy [26]. However, pioglitazone has also several concerns for wide clinical use, such as increased risks at prostate or pancreas cancer, body weight gain, fluid retention, bone fracture in women, and increased cardiovascular events. INT131, which is a selective PPARγ modulator (SPPARMγ), is in development for T2DM patients. INT131 demonstrated dose-dependent reductions in HbA1c, equivalent to 45 mg pioglitazone, but with less fluid accumulation and weight gain [27]. Although no study with INT131 for the NASH treatment has been initiated, its agent will be expected in the future. MSDC-0602K is a PPARγ-sparing thiazolidinedione. A study to evaluate the safety, tolerability, and efficacy of MSDC 0602K in patients with NASH is ongoing (EMMINENCE). This is a RDBPCT of three doses of MSDC-0602K or placebo given orally once daily to subjects with biopsy-proven NASH with fibrosis and no cirrhosis (NCT02784444).

**Fig. 3:**
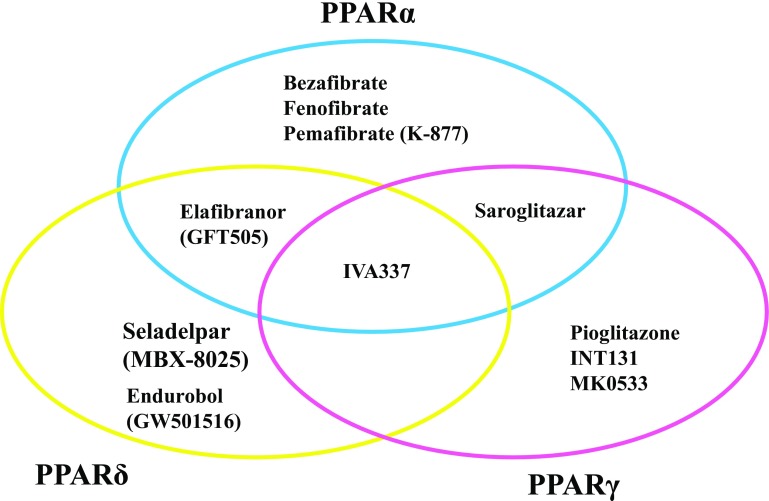
PPAR agonists for NASH/NAFLD

*PPARα* There have been no studies proving the efficacy of PPARα agonists, such as bezafibrate or fenofibrate, which are extensively used in the treatment of hypertriglyceridemia but have no impact in NASH/NAFLD. Bezafibrate has been believed to be effective in breast cancer patients with tamoxifen-induced NASH [28]. Pemafibrate (K-877), a selective PPARα modulator (SPPARMα), will be described as below.

*Saroglitazar* Saroglitazar is a dual PPARα/γ agonist approved for the treatment of dyslipidemia in diabetic patients in India [29]. Phase 2 RDBPCT comparing three doses of saroglitazar (1, 2, or 4 mg) with placebo in NAFLD is now ongoing (EVIDENCES II; NCT03061721). The primary endpoint of the study is percentage change from baseline in serum ALT levels at week 16 in the saroglitazar groups as compared to the placebo group.

*Elafibranor (GFT505)* Elafibranor is an unlicensed dual agonist of PPARα/δ receptors, and has been shown to improve steatosis, inflammation, and fibrosis in mouse models of NAFLD [30]. A phase IIb RDBPCT showed patients resolving NASH without worsening hepatic fibrosis with 120 mg elafibranor in those with NAS ≥ 4 (GOLDEN-505) [31]. Treatment was not effective in those with NAS < 4 (19 vs. 12%, *p* = 0.045). A multicenter, phase 3 RDBPCT study to evaluate the efficacy and safety of elafibranor in NASH without cirrhosis is ongoing (RESOLVE-IT) (NCT02704403). The primary objectives of this study are to evaluate the effect of elafibranor (120 mg/day) treatment in NASH patients (NAS ≥ 4) with stage 2/3 fibrosis compared to placebo on (1) histological improvement and (2) all-cause mortality and liver-related outcomes in patients with NASH and fibrosis. RESOLVE-IT is a RDBPCT (2:1), conducted in approximately 2000 patients, at 250 centers worldwide.

*Pan-PPAR agonist* IVA337 is an anti-fibrotic treatment with a unique mechanism of action going through the activation of all three alpha, gamma, and delta PPARs (pan-PPAR agonist). IVA337 is effective in experimental skin fibrosis and lung fibrosis [32, 33]. A phase 2b RDBPCT in NASH to assess IVA337 is now recruiting (NATIVE). NATIVE study will investigate the safety and efficacy of two doses of IVA337 (800 and 1200 mg/day) over a 24-week period and will enroll up to 225 patients in 12 European countries (NCT03008070).

#### Antidiabetic drugs

T2DM is strongly associated with NASH and liver-related mortality. The most important problem is what kinds of drugs are the most appropriate for NASH/NAFLD with diabetes among a variety of antidiabetic medications. Ideal anti-diabetic treatments on the view of NASH treatment must have weight-reducing efficacy, reduced cardiovascular event, prevention of HCC, low cost, and improved QOL [34]. There are no approved diabetic therapies except pioglitazone for NASH [5–8]. Metformin is now positioned as the first-line therapy according to the guideline of ADA/EASD because of its low cost, weight-reducing effect, preventive effect on cardiovascular event, and safety profiles. Unfortunately, metformin has no data regarding improvement in liver enzymes and histology in NASH/NAFLD, although it is associated with a reduced incidence of HCC and extrahepatic malignancies. Novel antidiabetic drugs will become a candidate for the treatment of NASH as we previously reviewed this point [34]. Incretin-associated drugs are classified into DPP4 inhibitors and glucagon-like peptide 1 receptor agonists (GLP-1RA). Unfortunately, there is conflicting evidence showing efficacy of DPP4 inhibitors in NASH/NAFLD patients with diabetes, although a number of patients involved into these studies is relatively small [34]. The efficacy of liraglutide, a GLP-1RA, was reported in NASH patients in Western countries (LEAN study [35]) and Japanese studies (LEAN-J study [36]). That most of patients naïve to injection therapy will hesitate daily injection therapy. Dulaglutide has some advantages such as weekly injection, disposable and prefilled device, and similar safety profiles to other GLP-1RAs. Semaglutide, a novel GLP-1 RA, is in advanced stages of development for diabetes. To investigate the effect of semaglutide on NASH, a phase 2 RDBPCT comparing the efficacy and safety of three different doses of once-daily subcutaneous semaglutide versus placebo in 372 participants with NASH is now ongoing (NCT02970942). Semaglutide has three advantages over other GLP-1RA. First, the SUSTAIN-6 trial showed that semaglutide has a potential benefit on prevention of cardiovascular events [37]. Second, semaglutide is superior to dulaglutide on glucose control and weight loss in T2DM patients (SUSTAIN 7 trial). SUSTAIN 7 is a phase 3b, 40-week, efficacy and safety trial of 0.5 mg semaglutide versus 0.75 mg dulaglutide and 1.0 mg semaglutide versus 1.5 mg dulaglutide, both once-weekly, as add-on to metformin in 1201 people with T2DM. Third, oral agent of semaglutide is now under development and of clinical use in the near future. As a result, among a variety of GLP-1 RA, dulaglutide or semaglutide will be the most promising in the treatment of diabetic NASH [34, 38]. According to the AASLD guidance, however, it is premature to consider GLP-1RA to specifically treat in NASH/NAFLD patients without diabetes [8] because of insufficient evidence. Sub-analyses of three RDBPCT of SGLT2 inhibitor (canagliflozin [39, 40], luseogliflozin [41]) for the treatment of T2DM, serum transaminase activities in SGLT2 inhibitor-treated patients were significantly reduced compared to those in the placebo group. The finding that causes of abnormal ALT level (31 IU/L or above) in a majority of Japanese diabetic patients may be associated with NAFLD [42] implies that the efficacy of SGLT2 inhibitor on NASH/NAFLD patients can be expected. Several pilot studies found significant reduction in ALT, body weight, and the fatty liver index in NAFLD patients [43–45]. The impact of SGLT2 inhibitor on liver histology is not confirmed. Takeda et al. reported a case of NASH with T2DM who resolved steatosis, inflammation, and hepatocyte ballooning after the ipragliflozin treatment [46]. Akuta et al. also recently demonstrated that all eight NAFLD patients with SGLT2 administration relieved hepatic steatosis and three of them obtained improvement in liver fibrosis [47]. Two open RCTs have been reported from Japan to compare the efficacy of SGLT2 inhibitor to other diabetic medications such as pioglitazone and metformin. The first repot is to compare the effect of luseogliflozin to metformin in T2DM patients with NAFLD. Hepatic steatosis, evaluated by liver-to-spleen (*L*/*S*) ratio on CT, was significantly reduced in the luseogliflozin group compared to in the metformin group [48]. The aim of another report is to compare the efficacy of ipragliflozin versus pioglitazone in NAFLD patients with T2DM. Serum ALT levels, HbA_1c_, and fasting plasma glucose were similarly reduced in the two treatment groups. Nevertheless, body weight and visceral fat area showed significant reductions only in the ipragliflozin group compared with the pioglitazone group [49]. A few open pilot studies of SGLT2 inhibitor in NAFLD patients are ongoing in the western countries (NCT02696941) or Asia (NCT02875821, NCT02964715). The effect of SGLT2 inhibitors versus other diabetic drugs (metformin, sulfonyl urea) is also investigated (NCT02696941, NCT02649465). The effects of empagliflozin treatment on hepatocellular lipid content, liver energy metabolism, and body composition is now investigated in a multicenter, RDBPCT, interventional, and exploratory pilot study in patients with newly diagnosed T2DM (NCT02637973).

#### Lipid-altering agents

*Approved agents for dyslipidemia* Ezetimibe, a potent inhibitor of cholesterol absorption, has been explored for the treatment of NASH/NAFLD, but conflicting results exist [50–52]. Histological findings (steatosis and inflammation) have been relived after the ezetimibe treatment without control arms [50, 51]. A RDBPCT (MOZART study) showed that ezetimibe 10 mg orally daily for 24 weeks did not significantly affect hepatic steatosis over placebo [52]. A meta-analysis using six studies (two RCT and four pilot) has shown that ezetimibe may decrease serum liver enzymes and hepatic steatosis, but histological effectiveness remains uncertain [53]. Omega-3 fatty acids are often administrated for patients with hypertriglyceridemia. Two large studies (EPE-A study [54], WELCOME study [55]) failed to show the therapeutic benefit of omega-3 fatty acids in patients with NASH/NAFLD. Omega-3 fatty acids is limited to be used in NASH/NAFLD with hypertriglyceridemia [8]. Since NAFLD patients are at high risk of cardiovascular morbidity or mortality, statins could be used to treat dyslipidemia with NASH/NAFLD [8]. Statin use seems to be associated with inhibition of hepatic inflammation, improvement of hepatic fibrosis, and reduced hepatocarcinogenesis [56], although prospective RCTs are now difficult to perform.

*Pemafibrate* Pemafibrate, a novel SPPARMα, was approved in Japan in 2017. In Japan, phase 2, RDBPCT decreased serum transaminase activities as well as lipid profiles in patients with dyslipidemia without increasing adverse effects [57]. Pemafibrate, which improves liver pathology in diet-induced rodent model of NASH [58], will become a promising therapeutic agent for human NASH. In Japan, clinical trials for the treatment of NAFLD/NASH will begin in the near future.

*Aramchol* Aramchol, a cholic-arachidic acid conjugate, has inhibitory effects of stearoyl-CoA desaturase (SCD). Aramchol was initially produced for treatment of gallstone [59]. However, animal experiments showed a strong reduction of hepatic fat accumulation rather than gallstone dissolution. In humans, hepatic fat content was significantly reduced in the aramchol (300 mg/day) group [60]. Higher doses of aramchol (400 and 600 mg) are currently being tested on biopsy-proven NASH patients without cirrhosis in a 52-week phase 2b trial, which evaluates their effect on hepatic triglyceride content using MR spectroscopy (NCT02279524).

*GS0976* Acetyl-CoA carboxylase (ACC) is a key enzyme that regulates the conversion of malonyl-CoA to acetyl-CoA [61]. Malonyl-CoA is a key regulator of fatty acid metabolism, controlling the balance between de novo lipogenesis and fatty acid oxidation. An open-label, proof-of-concept study evaluating GS-0976, an investigational inhibitor of ACC, in NASH patients. The data, from ten patients treated with GS-0976 20 mg taken orally once daily for 12 weeks, indicated that treatment was associated with statistically significant improvements in liver fat content and noninvasive markers of fibrosis (NCT02856555). At week 12, patients receiving GS-0976 experienced a 43% median relative decrease in liver fat content, from 15.7 to 9.0% (*p* = 0.006), as measured by magnetic resonance imaging-proton density fat fraction (MRI-PDFF). Median liver stiffness, a noninvasive marker of fibrosis, declined from 3.4 to 3.1 kPa at week 12 (*p* = 0.049), as assessed by magnetic resonance elastography (MRE). In addition, patients with reductions in hepatic fat demonstrated improvements in liver biochemistry and serum markers of fibrosis and apoptosis, supporting the biological activity of GS-0976. A separate phase 2 RDBPCT evaluating GS-0976 in 126 patients with NASH is completed. According to Liver Meeting 2017, GS-0976 demonstreated signficant decrease in hepatic fat content and TIMP-1 (a serum marker associated with hepatic fibrosis).

#### Anti-hypertensive drugs

*Angiotensinogen receptor blockers (ARB)* There are no particularly favored agents for control of hypertension, although a few studies suggest that angiotensinogen receptor blockers (ARB) may have anti-fibrotic effects in NASH patients [62, 63]. Unfortunately, a RDBPCT regarding the effect of losartan for 96 weeks in NASH patients failed because of slower recruitment than expected due to the widespread use of ARB in NASH patients [64]. It may be difficult to plan prospective RDBPCT to establish the efficacy or ARB for the treatment of NASH/NAFLD.

#### FXR ligand

*Obeticholic acid (OCA)* Obeticholic acid (OCA), a ligand of farnesoid X receptor (FXR), is a synthetic variant of natural bile acid chenodeoxycholic acid. In animal models, FXR activation has been demonstrated to reduce hepatic glucogenesis, lipogenesis, and steatosis. In the FLINT trial, treatment with OCA achieved a primary end-point of improving the necro-inflammation without worsening of fibrosis in 46% of the treated patients with NASH. Moreover, compared to placebo, NASH resolution was obtained in 22% of treated patients [65]. A phase 2 RDBPCT in Japan (FLINT-J trial) showed that high doses of OCA (40 mg/day) significantly resolved NASH compared with placebo (38 vs. 20%, *p* = 0.049). Fibrosis improvement in the OCA treated group is similar to that in the placebo group. There are plausible reasons explaining this discrepancy between FLINT and FLINT-J study. In the FLINT-J study, NASH with mild fibrosis at entry is prevalent. Some patients in the OCA group refused post-treatment liver biopsy, and those are classified into non-responders. An international, phase 3 study (REGENERATE study) is now ongoing. However, OCA has several drawbacks, such as elevated LDL levels, itching, and high cost [65].

*INT-767* INT-767 is a bile acid analogue that acts as a dual agonist on FXR/Takeda G-protein-coupled receptor 5 (TGR5). In an animal model, INT-767 improved histological features of NASH and modulated the activation of hepatic monocytes [66]. TGR5 has been known to affect energy metabolism, glucose homeostasis, bile composition/secretion, and inflammation.

*Non-bile acid FXR* Selective non-bile acid synthetic FXR agonists have been developed to resolve disadvantage of OCA. Those have the potential to provide metabolic effects without increasing side effects of pruritus and elevated LDL. Phase 2 studies with GS-9674 are ongoing in patients with NASH (NCT02854605), primary biliary cholangitis (PBC), and primary sclerosing cholangitis (PSC). Two other FXR agonists, LMB763 (NCT02913105) and LJN452 (NCT02855164), have been developed and are in phase 2 trials.

*MGL-3196* The thyroid hormone receptor β (THRβ) is the predominant liver thyroxine (T4) receptor, through which increased cholesterol metabolism and excretion through bile is mediated. MGL-3196, a highly selective THRβ agonist, has been developed to target dyslipidemia but has also been shown to reduce hepatic steatosis in fat-fed rats [67]. Phase 2 trials are ongoing in patients with biopsy-proven NASH and ≥ 10% liver steatosis using percent change from baseline hepatic fat fraction assessed by MRI-PDFF as a primary outcome (NCT02912260).

#### Anti-inflammatory and anti-apoptosis agents

*Pentoxifylline* Pentoxifylline (PTX), a methylxanthine derivative, has anti-inflammatory effects and decreases oxidative stress. An RDBPCT showed that PTX therapy for 1 year significantly improved histological features of NASH compared to placebo [68]. A meta-analysis evaluating five studies showed improvement histological findings such as lobular inflammation and NAS without affecting lipid profiles [69]. However, this drug is no longer commercially available in Japan because its initial efficacy for treating after-effects of brain stroke was reevaluated and found to be insufficient.

*Selonsertib* Apoptosis signal-regulating kinase 1 (ASK1) is activated by extracellular TNFα, intracellular oxidative or ER stress and initiates the p38/JNK pathway, resulting in apoptosis and fibrosis [70]. Inhibition of ASK1 has therefore been proposed as a target for the treatment of NASH. An open-label phase 2 trial evaluating the investigational ASK1 inhibitor selonsertib (GS-4997) alone or in combination with the monoclonal antibody simtuzumab (SIM) in NASH patients with moderate-to-severe liver fibrosis (stages 2/3). The data demonstrate regression in fibrosis that was, in parallel, associated with reductions in other measures of liver injury in patients treated with selonsertib for 24 weeks. Patients receiving selonsertib demonstrated improvements in several measures of liver disease severity, including fibrosis stage, progression to cirrhosis, liver stiffness (measured by MRE), and liver fat content (measured by MRI-PDFF). As no differences were observed between combination and monotherapy, results are presented for selonsertib (18 and 6 mg) with/without SIM and for SIM alone [71]. Thus, international phase 3 trials evaluating selonsertib among NASH patients with stage 3 (STELLAR3; NCT03053050) or cirrhosis (STELLAR4; NCT03053063) are ongoing (STELLAR program).

*Tipelukast* MN-001 (tipelukast) is a novel, orally bioavailable small-molecule compound that exerts its effects through several mechanisms to produce its anti-fibrotic and anti-inflammatory activity in preclinical models, including leukotriene (LT) receptor antagonism, inhibition of phosphodiesterases (PDE) (mainly 3 and 4), and inhibition of 5-lipoxygenase (5-LO). An open-label study to evaluate the efficacy, safety, tolerability, and PK of MN-001 (Tipelukast) on HDL function and serum triglyceride levels in NASH/NAFLD with hypertriglyceridemia is ongoing (NCT02681055).

*Emricasan* Emricasan, an irreversible caspase inhibitor, improves NAS and fibrosis in murine models of NASH [72]. A phase 2b study in patients with NASH (stage 1-3) is evaluating the efficacy of 72 weeks of emiricasan 10 or 100 mg (ENCORE-NF, NCT02686762). Another phase 2b study in patients with NASH with cirrhosis and severe portal hypertension is assessing the efficacy of three doses Emricasan (10, 50, 100 mg/day) on portal hypertension (ENCORE-PH, NCT02960204). Primary outcome is mean change in hepatic venous pressure gradient (HVPG).

*Vascular adhesion protein-1 (VAP-1) inhibitor (BI 1467335)* The adhesion molecule vascular adhesion protein-1 (VAP-1) is a membrane-bound amine oxidase that promotes leukocyte recruitment to the liver, and the soluble form (sVAP-1) accounts for most circulating monoamine oxidase activity, has insulin-like effects, and can initiate oxidative stress [73]. VAP-1 is directly involved in stellate cell activation and is a strong profibrogenic stimulus. Thus, targeting VAP-1 may result in a decrease in leukocyte recruitment and reduction of inflammation and fibrosis. BI 1467335 is a VAP-1 inhibitor that works by blocking leucocyte adhesion and tissue infiltration in inflammatory process. Phase 2a trial of BI 1467335 is a multicenter, RDBPCT in 150 patients with clinical evidence of NASH (NCT03166735).

#### Gut microbiome

*IMM-124e* IMM-24e is an IgG-rich extract to bovine colostrum from cows immunized against lipopolysaccharide (LPS). IMM-24e can reduce exposure of the liver to gut-derived bacterial products and LPS. An open-label, phase 1/2 clinical trial in ten patients with biopsy-proven NASH improved liver enzymes as well as glycemic control via increase in serum levels of GLP-1, adiponectin, and T regulatory cells [74]. A phase 2 RDBPCT of IMM-124E for 24 weeks is currently ongoing for NASH patients (NCT02316717).

*Solithromycin* Solithromycin is a highly potent next-generation macrolide antibiotic. In a phase 2 open-label study, all six NASH patients had reductions in NAS (mean reduction, 1.3) and ALT level (mean reduction, 17.8 U/L) after 90 days of treatment with solithromycin (NCT02510599).

*TLR4 antagonist* JKB-121 is a long-acting small molecule that is efficacious as a weak antagonist at the Toll-like receptor 4 (TLR4). It is a non-selective opioid antagonist that has been shown to prevent the LPS-induced inflammatory liver injury in a methionine/choline-deficient diet fed rat model of NAFLD. In vitro, JKB-121 neutralized or reduced the LPS-induced release of inflammatory cytokines, deactivated hepatic stellate cells, inhibited hepatic stellate cell proliferation, and collagen expression. Inhibition of the TLR4 signaling pathway may provide an effective therapy in the prevention of inflammatory hepatic injury and hepatic fibrosis in NASH patients [75]. A phase 2 RDBPCT trial of JKB-121 for the treatment of NASH is ongoing (NCT02442687).

#### Antifibrotic agents

Given that hepatic fibrosis stage is the most important determinant of mortality in NASH patients [9, 76], there is an unmet medical need for an effective anti-fibrotic treatment for those with advanced fibrosis. Several anti-fibrotic agents have been developed for the treatment of advanced NASH.

*Cenicriviroc* Cenicriviroc (CVC), a C–C motif chemokine receptor-2/5 (CCR2/5) antagonist, has been developed to primarily target inflammation. This agent has also antifibrotic effects and improves insulin sensitivity. Macrophage recruitment through CCR2 into adipose tissue is believed to play a role in the development of insulin resistance and T2DM. Administration of CCR2 antagonist resulted in modest improvement in glycemic parameters compared with placebo [77]. CCR5 antagonist is expected to impair the migration, activation, and proliferation of collagen-producing hepatic stellate cells [78]. According to phase 2b trial (CENTAUR study), significant improvement of fibrosis without worsening NASH after 1 year of CVC treatment was found (20%) compared with placebo (10%) [79]. Although asymptomatic amylase elevation (grade 3) was more frequent in the CVC group than in the placebo group, this agent is well tolerated. Phase 3 evaluation for the treatment of NASH with stage 2/3 fibrosis is now ongoing and recruiting (AURORA study; NCT03028740).

*Simtuzumab (SIM)* SIM is a monoclonal antibody against the enzyme lysyl oxidase-like 2 (LOXL-2) responsible for the cross-linking of collagen and overexpressed during the fibrosis progression [80]. Unfortunately, this agent could not bring additional benefit over ASK1 inhibitor to improve hepatic fibrosis in phase 2b study as mentioned above. Finally, SIM was withdrawn from candidates of NASH treatments.

*Galectin-3 antagonist* Galectin-3 protein expression, which is essential to the development of hepatic fibrosis, was increased in NASH with the highest expression in macrophages surrounding lipid-laden hepatocytes. In mice models, GR-MD-02, a galectin-3 inhibitor, resulted in marked improvement in liver histology with a significant reduction in NASH activity and collagen deposition [81]. Although there was no safety concern in phase 2a trials in NASH patients with stage 3 fibrosis [82], there was no apparent improvement in the three non-invasive tests for assessment of liver fibrosis. A phase 2b clinical trial to evaluate the safety and efficacy of GR-MD-02 for the treatment of liver fibrosis and resultant portal hypertension in 162 patients with NASH cirrhosis (NASH-CX trial) is now ongoing (NCT02462967). Top-line results will be reported in early December 2017.

*ND-LO2-s0201* Hsp47 (heat shock protein 47) is a collagen-specific molecular chaperone that is essential for the maturation and secretion of collagen. ND-LO2-s0201 is a vitamin A-coupled lipid nanoparticle containing siRNA against HSP47. A phase 1 open study is completed to evaluate in subjects with severe hepatic fibrosis (stage 3/4) (NCT02227459).

#### Ongoing or scheduled clinical trials in Japan

To our best knowledge, there are several ongoing or scheduled clinical trials in Japan. Two agents, including semaglutide and ASK1 inhibitor, were already mentioned above.

*Nonsteroidal MRA* Several nonsteroidal antagonists of the mineralocorticoid receptor (MRA) are in clinical development with a clear focus on the treatment of diabetic kidney diseases. In Japan, MT3995, a novel nonsteroidal MRA, is currently tested for the treatment of NASH (phase 2, NCT02923154).

*FGF-21 (fibroblast growth factor-21)* Fibroblast growth factor 21 (FGF-21), a hepatokine, is a 181-amino-acid-secreted protein that is produced in the liver. FGF-21 regulates glucose in the liver and the white adipose tissue and its circulating levels are elevated in NAFLD patients, considered to play a protective role against NAFLD [83]. A RCT in a small group of obese T2DM patients with FGF-21 found significant improvement in lipid profiles as well as weight loss, reduced insulin levels, and raised adiponectin [84]. A phase 2 study of BMS-986036, a recombinant FGF-21 in NASH patients for 16 weeks, is completed (NCT02413372). This was a multicenter RDBPCT (1:1:1) in adults with BMI ≥ 25 kg/m^2^, biopsy-proven NASH with stage 1–3, and hepatic fat fraction ≥ 10%, assessed by MRI-PDFF. Patients received subcutaneous injections of BMS-986036 10 mg daily (*n* = 25), BMS-986036 20 mg weekly (*n* = 23), or placebo (*n* = 26) daily for 16 weeks. The primary efficacy endpoint was absolute change in MRI-PDFF at week 16. At week 16, both dosing regimens of BMS-986036 (10 mg daily or 20 mg weekly) significantly reduced liver fat as measured by MRI-PDFF versus placebo (6.8 and 5.2%, respectively, Z. 1.3%, *p* = 0.0004 and *p* = 0.008). Both dosing regimens also improved Pro-C3 (N-terminal type III collagen propeptide, a fibrosis biomarker [85]), liver stiffness evaluated by MRE, as well as adiponectin, ALT, and AST. Improvements in lipid profiles were also observed in the treatment groups. Overall, BMS-986036 had a favorable safety profile, with no deaths or serious adverse events related to treatment, and no discontinuations due to adverse events. An international phase 3 study of BMS-986036 for the treatment of NASH with stage 3/4 will be planned.

#### Drug repositioning

*Amlexanox* Amlexanox is an inhibitor of noncanonical IκB kinases IKK-ɛ and TANK-binding kinase 1. Amlexanox is an approved small-molecule therapeutic presently used in the clinic to treat aphthous ulcers and asthma. Treatment of obese mice with amlexanox elevates energy expenditure through increased thermogenesis, producing weight loss, improved insulin sensitivity, and decreased steatosis. Because of its record of safety in patients, amlexanox may be an interesting candidate for clinical evaluation in the treatment of NAFLD [86]. An open-label study and a phase 2 RDBPCT are currently assessing the effects of 12 weeks of amlexanox in patients with diabetes, obesity, and fatty liver on hepatic fat content by MRI, HbA1c, and weight (NCT01975935 and NCT01842282).

*Pirfenidone* Pirfenidone (PFD) is an orally bioavailable pyridone derivative that has been clinically used for the treatment of idiopathic pulmonary fibrosis [87]. PFD markedly attenuated liver fibrosis in Western diet (WD)-fed melanocortin 4 receptor-deficient (MC4R-KO) mice without affecting metabolic profiles or steatosis. PFD prevented liver injury and fibrosis associated with decreased apoptosis of liver cells in WD-fed MC4R-KO mice [88]. PFD can be repositioned as an antifibrotic drug for human NASH.

### Milestones in the treatment of NASH/NAFLD

Until now, the gold standard of assessment for treatment efficacy in NASH has been liver histology. However, repeated liver biopsies are practically difficult to be performed in NASH patients, because of risk, sampling error, observers’ variability of pathological interpretation, and cost. Simple, reliable, and cost-effective parameters should be established to monitor the disease and evaluate the treatment efficacy.

*ALT* In the sub-analysis of PIVENS study, ALT response, which was defined as ALT reduction over 30% from baseline or ALT levels less than 40 IU/l, reflect histological improvement [89]. As we also previously reported in 2015 [90], ALT response was the best predictor of reduction in NAS or fibrosis regression in 52 Japanese patients with NASH undergoing repeated biopsies.

*Body weight* Weight loss has been believed to be associated with improvements of liver histology in patients with NAFLD/NASH. Data from 261 NASH patients receiving repeated liver biopsies showed that weight loss, the absence of diabetes, ALT normalization, and baseline NAS less than 5 were independent predictors of NASH resolution without fibrosis worsening after 1 year of lifestyle intervention [91].

**Fig. 4:**
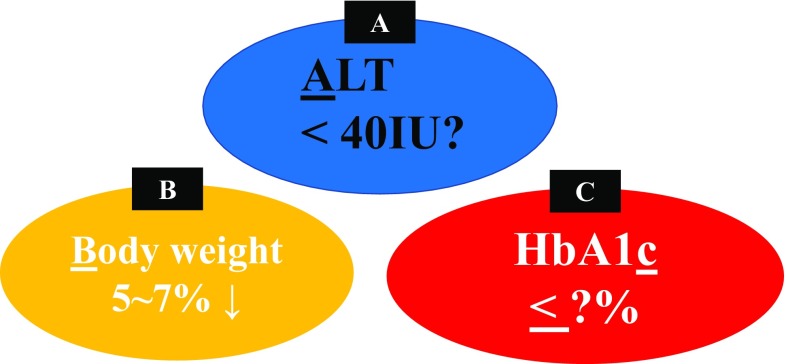
Milestones in the treatment of NASH are “ABC”??

*HbA1c* Decreased levels in HbA1c [92] were more strongly associated with fibrosis improvement in 39 Japanese patients with diabetes and NAFLD who underwent sequential liver biopsies. As a result, we believe that these three clinical parameters, including ALT, body weight, and HbA1c (ABC), can become the milestones in the treatment of NASH (Fig. 4), although the appropriate goal of each parameter to ameliorate hepatic fibrosis will be established.

*FIB4 index* Fibrosis 4 (FIB-4) index and NALD fibrosis score (NFS) are now established as the best predictors of severe fibrosis in NAFLD [8, 93–95]. FIB-4 index is simply calculated using an algorithm based on AST, ALT, platelet count, and age. The FLINT study demonstrated that OCA treatment of NASH patients led to a statistically significant decrease in FIB4 index from baseline as compared to placebo (OCA: − 0.246 vs. placebo: − 0.047; *p* = 0.0076). Further, a decline in FIB-4 of 10% after 24 weeks of treatment predicted improvement in fibrosis by at least one stage as assessed by biopsy at 72 weeks (*p* = 0.0448). While NFS declined in the OCA-treated patients and increased in the placebo patients, it did not appear to be sensitive to changes in fibrosis. This result, presented by Dr. Sanyal in the annual meeting of AASLD held in 2015, provides support for the use of FIB-4 index as potential non-invasive alternative means for monitoring fibrosis changes in response to treatment (Fig. 4).

*Imaging* Although imaging studies such as VCTE (Fibroscan) [96–98] and MR elastography (MRE) [99] have been extensively studied to detect severe fibrosis in NAFLD [8, 95], it is unknown whether these modalities are also useful to evaluate treatment efficacy. Many ongoing clinical trials, which evaluate clinical efficacy of NASH/NAFLD, are using imaging modalities using MRI-PDFF/MRE, which will become an alternative to liver biopsy.

**Fig. 5:**
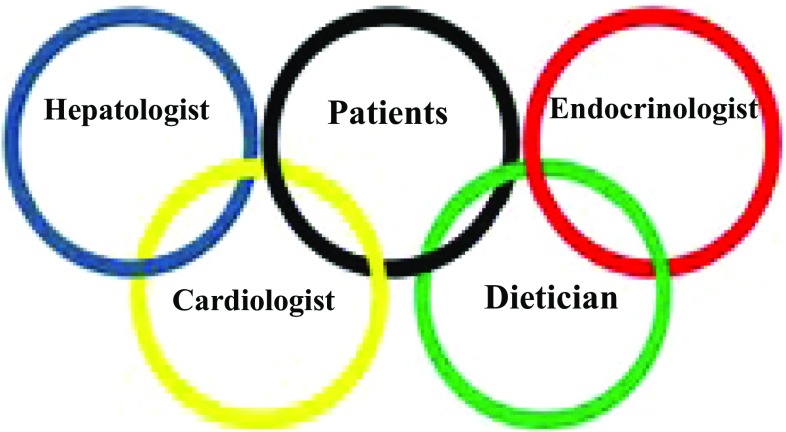
Variety of stakeholders in the treatment of NASH/NAFLD

### Who should treat?

Diabetes specialist should pay attention to liver status. Three studies using Fibroscan showed that 12–18% of diabetic patients are estimated to have significant liver fibrosis by different cutoffs [100–102]. Another study using MRE found that advanced fibrosis (defined as MRE ≥ 3.6 kPa) was 7.1% in diabetic patients [103]. In a cross-sectional multicenter study conducted by JSG-NAFLD, the presence of diabetes is associated with advanced fibrosis in 1365 biopsy-proven NAFLD patients [104]. These results imply the importance of collaboration between the hepatologists and diabetes team. However, the leading cause of mortality in patients with NAFLD is cardiovascular diseases, followed by extrahepatic cancer and liver-related diseases [105]. There are a variety of stakeholders in the treatment of NASH/NAFLD, including the hepatologist, cardiologist, endocrinologist, dietician, and patients (Fig. 5). Lifestyle modification intervention and pharmacotherapies should be delivered in collaboration with multi-disciplinary medical staff [106] (Fig. 5).

**Fig. 6:**
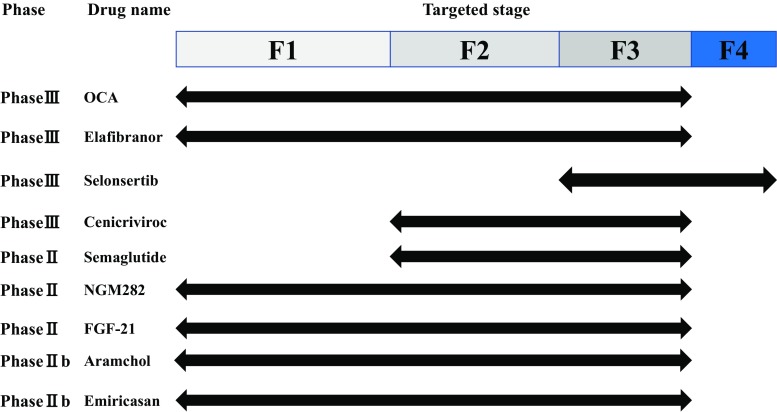
NASH drug pipelines

### Future perspectives

Clinically meaningful outcomes in patients with NAFLD/NASH are shown in Table 2. The primary endpoint should be measurable, sensitive to change, clinically meaningful, and be able to be quantified consistently. Reversal of steatohepatitis with at least no worsening of fibrosis has been considered as a primary endpoint of clinical trials.

**Table 2:** Clinically meaningful outcomes in patients with NAFLD/NASH

Histological improvement in NASH
Steatosis
Inflammation
Hepatocyte ballooning
Fibrosis
Incident neoplasms
Hepatic cancer
Extra-hepatic cancer
Mortality/morbidity
All cause mortality/morbidity
Liver—related mortality/morbidity (LT)
Symptoms/QOL (patient reported outcome: PRO)
Hepatic failure (ascites, edema, jaundice, variceal hemorrhage, etc.)
QOL (physical health, mental health)
Labor productivity
Well-being
Economics
Lifetime medical cost

Although hepatic fibrosis is the most critical determinant of all-cause or liver-related mortality in NASH, it remains to be solved whether improvement in hepatic fibrosis can lead to prevention of liver-related mortality in NASH patients. The prevalence of obesity and diabetes is dramatically increasing worldwide. NASH-related liver diseases (HCC, hepatic failure, and variceal hemorrhage) will soon be the leading causes of liver transplantation. It is estimated that the market size of NASH will reach 49 billion in 2027 in US, Japan, and EU 5 (England, France, Germany, Italy, and Spain). When pharmacotherapies are initiated for the target of liver disease in NASH/NAFLD, benefits and risks should be discussed with patients [8]. Patient-reported outcome (PRO) should be evaluated before and after pharmacotherapies. It is estimated that the risk for HCC development in NASH/NAFLD without advanced fibrosis is very small given the extremely large number of patients without advanced fibrosis within the general population. Economical cost and benefit should also be balanced [107].

## Conclusions

To prevent liver-related morbidity/mortality in NASH patients, those with fibrosis should be considered for pharmacotherapies in addition to conventional dietary interventions. The first-line therapy for those without diabetes is vitamin E on the basis of accumulating evidence, although its impact on preventive effect of hepatic fibrosis and hepatocarcinogenesis remain uncertain. Diabetic NASH patients should be preferentially treated with novel drugs licensed for diabetes treatment such as GLP-1RA and SGLT2 inhibitors. SPPARMα (pemafibrate) is promising in NASH patients with dyslipidemia. There are currently several innovative agents in the drug pipeline for NASH worldwide. Four drugs (OCA, elafibranor, selonsertib, and CVC) have entered phase 3 trials (Fig. 6). Cost-effectiveness data and patient-centered benefits are also required to position their medications in the practical guidelines of NASH/NAFLD.
